# The pubic midline exposure for symphyseal open reduction and plate fixation

**DOI:** 10.1007/s10195-014-0296-9

**Published:** 2014-05-08

**Authors:** Mark R. Adams, John A. Scolaro, Milton Lee Chip Routt

**Affiliations:** 1 Department of Orthopaedics, Rutgers, New Jersey Medical School, 140 Bergen Street, D level, Newark, NJ 07103 USA; 2 Irvine Department of Orthopaedic Surgery, University of California, 101 The City Drive South Pavillion III, Building 29A, Orange, CA 92868 USA; 3 Department of Orthopaedic Surgery, The University of Texas, Health Sciences Center at Houston, 6431 Fannin Street, Houston, TX 77030 USA

**Keywords:** Pelvic ring, Pfannenstiel, Symphysis approach

## Abstract

**Background:**

Open reduction and plate fixation of the disrupted symphysis pubis is commonly performed through a horizontal Pfannenstiel incision. Certain clinical situations that complicate the soft tissue conditions of the lower abdomen may make the Pfannenstiel incision a less appealing option. We report on the use of a vertical pubic area midline skin incision in a series of patients undergoing open reduction and plate fixation of their traumatically disrupted symphysis pubis.

**Materials and methods:**

Institutional Review Board approval was obtained for a retrospective chart review of the charts of 25 patients treated between September 2011 and October 2012. Their charts were reviewed for patient age, gender, body mass index (BMI), pelvic injury type (as classified by Young and Burgess), mechanism of injury and associated traumatic injuries. The depth of the approach was estimated using the pelvic computed tomography (CT) scan. Details from the operative procedure were recorded, as was the length of follow-up and any perioperative complications.

**Results:**

Twenty-five patients were eligible for inclusion during the defined study time period between September 2011 and October 2012. The patients’ average age was 55.8 years (range 25–91). All patients were males. The average BMI was 29.3 (range 18.8–43.8). The depth measured on the axial pelvic CT scan from skin to symphysis was 57.6 mm (range 35.2–90.2 mm). Five of 25 patients had an isolated pelvic ring injury without other associated injuries. The injury pattern was APC2 in 18, APC3 in 3, LC2 in 2, LC3 in 1 and VS in 1 patient(s) [anterior posterior compression (APC), lateral compression (LC), vertical shear (VS)]. Urologic procedures were performed in the same surgical setting in four patients. The average blood loss was 244 ml (range 150–400 ml). The average follow-up was 2.5 months (range 1–12 months). Perioperative issues were noted in two patients. One patient died within a month of surgery as a result of his associated traumatic injuries. One patient developed a deep infection.

**Conclusion:**

The pubic midline skin exposure is a feasible alternative to the Pfannenstiel incision for open reduction and plate fixation of the pubic symphysis.

**Level of evidence:**

IV, Retrospective case series

## Introduction

Open reduction and plate fixation has become a common treatment method for complete traumatic pubic symphyseal disruptions. The horizontal Pfannenstiel incision located slightly cranial to the palpable pubis provides access to the disrupted symphysis and anterior pelvic ring. This surgical exposure is also described for both the Stoppa exposure and as the “medial window” of the ilioinguinal surgical approach [1–5].

**Fig. 1:**
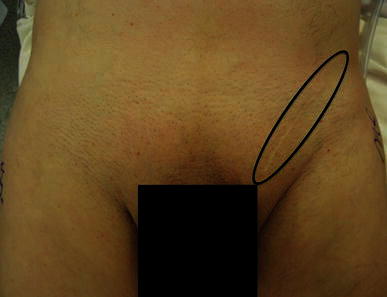
Clinical photograph of patient who had a previous left inguinal hernia surgery. A low midline approach was chosen for this patient to avoid the prior surgical scar

**Fig. 2:**
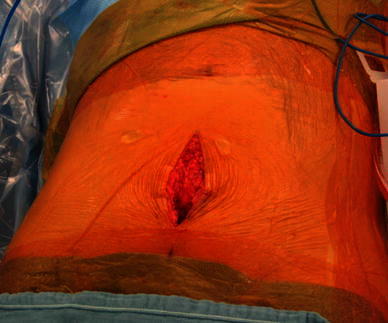
Intraoperative photograph showing low vertical skin incision used for midline approach to the anterior ring and pubic symphysis

In certain clinical scenarios, the location of the Pfannenstiel transverse incision may not be a practical option for the surgeon for numerous reasons. Open traumatic or surgical wounds, previously complicated but healed surgical scarring, or local skin conditions may preclude a horizontal incision along the lower abdomen (Fig. 1). We report the use of a pubic midline skin incision for the operative fixation of the pubic symphysis (Fig. 2).

Hirvensalo et al. [7] reported the use of either a low midline incision or Pfannenstiel in a series of 120 patients who had pelvic or acetabular fractures and were treated with surgery. The study describes the view available to the surgeon through the pubic midline incision but does not report any specifics on complications or limitations of the exposure. Nor does the paper report the exact number of patients treated with this approach.

We performed a retrospective clinical review of the pubic midline exposure in a consecutive series of adult patients with traumatic and complete symphysis pubis disruptions treated with open reduction and plate fixation. The purpose was to determine whether this was a viable alternative to the Pfannenstiel incision, with a particular focus on patient issues in the perioperative period. This is the first series of patients in the literature to have been treated with this incision and had their results examined [7].

## Materials and methods

Institutional Review Board approval was obtained for this study. The charts of 25 adult patients treated with open reduction and plate fixation of the anterior pelvic ring via a pubic midline approach between September 2011 and October 2012 were identified. The anterior pelvic ring pathology in these 25 patients involved isolated complete symphyseal disruptions; there were no concomitant rami, iliac or acetabular injuries.

**Fig. 3:**
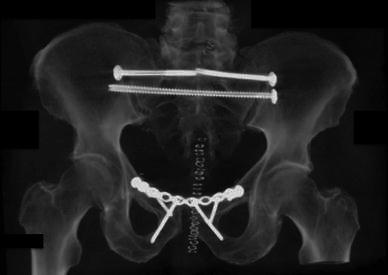
Three-dimensional reconstruction image from the postoperative CT scan of a patient with a pelvic ring injury who had pubic symphysis fixation with a 10-hole 3.5-mm pelvic reconstruction plate placed through a vertical midline incision

This was a consecutive series of patients treated with a traumatic disruption to the symphysis pubis whose injuries were treated operatively. Patients were admitted to the general surgery trauma service and taken to the operating room after clearance was obtained, usually within the first day of admission. Fifteen pounds of distal femoral traction was applied to those with complete posterior ring injuries. This was typically maintained during the operation and removed at the conclusion of the procedure. The patients were placed supine on a radiolucent flat-top table, and their pelvis was prepped and draped in standard fashion. The incision started approximately 1 cm proximal to the symphysis pubis at the midline, and extended proximally 8 cm. The midline rectus abdominus muscular raphe was then opened, and the space of Retzius was identified. The hematoma was removed, and the bladder was retracted using a malleable retractor. The rectus abdominus was retracted anteriorly and laterally on each side. Application of reduction clamps and insertion of 3.5 reconstruction plates proceeded in a routine manner. An 8-hole 3.5-mm pelvic reconstruction plate (Zimmer Holdings Inc., Warsaw, IN, USA) was used in 23 patients while a 10-hole 3.5-mm pelvic reconstruction plate was used in 2 patients (Fig. 3).

All information was obtained from physician documentation from the time of initial presentation to the most recent encounter. Patient information obtained included: age, gender, body mass index (BMI), pelvic injury type (as classified by Young and Burgess [6]), mechanism of injury and associated traumatic injuries. The depth of the approach was estimated using the pelvic computed tomography (CT) scan. The pelvic CT scan, consisting of axial, coronal, and sagittal images was generated with a spiral scanner, and was composed of 2.5-mm intervals between axial images (General Electric (GE) Company LightSpeed Series Scanner, Fairfield, CT, USA). The most cranial axial image that allowed for visualization of the right and left pubis at the level of the symphysis was selected, and the distance (in millimeters) from the skin to the anterior aspect of the pubic symphysis was measured and recorded for each patient (GE PACS, Fairfield, CT, USA). Blood loss (in milliliters) of the surgical procedure, involvement of urologic and/or general surgeons, as well as details of the orthopaedic implant used to fix the disrupted pubic symphysis, were recorded. Finally, length of follow-up and wound complications were noted for all patients.

## Results

The 25 patients were treated during the defined study time period between September 2011 and October 2012 (Table 1). The patients’ average age was 55.8 years (range 25–91). All patients were male. The average BMI was 29.3 (range 18.8–43.8). The depth measured on the axial pelvic CT scan from skin to symphysis was 57.6 mm (range 35.2–90.2 mm). The mechanism of injury varied but involved a motorcycle/motor vehicle collision or fall from >15 ft in the majority of patients. Six of 25 patients had an isolated pelvic ring injury without other associated injuries. The injury pattern, as described by Young and Burgess, was APC2 in 18, APC3 in 3, LC2 in 2, LC3 in 1 and VS in 1 patient(s). Eleven patients sustained associated long-bone fractures. Four patients required concomitant urologic procedures at the time of their orthopaedic pelvic surgery. Three patients had a repair of an extraperitoneal bladder rupture and one patient had an irrigation, debridement, and closure of his scrotal laceration. The average surgical blood loss (including the urological surgery procedure portions) was 244 ml (range 150–400 ml).

**Table 1:** Patient list

Patient	Age	Sex	BMI	CT depth (mm) to symphysis	Injury^a^	Mechanism	EBL (cc)	GU surgery	Complications in follow-up	Associated injuries present
1	65	M	30	55.26	APC3	Motorcycle collision	300	None	No complications	Yes
2	51	M	34.3	90	APC2	Motorcycle collision	300	Extraperitoneal bladder repair	Deep infection	Yes
3	65	M	29.4	50.95	APC2	Fall 15 ft	200	None	No complications	Yes
4	44	M	31.2	59.83	APC2	Pedestrian struck by vehicle	200	None	No complications	Yes
5	66	M	43.8	67.24	APC2	Motorcycle collision	300	None	No complications	Yes
6	82	M	24.8	41.21	APC2	Equestrian	250	None	No complications	Yes
7	27	M	29.4	54.9	APC2	Motorcycle collision	200	None	No complications	Yes
8	80	M	26.8	42.7	APC2	Fall 10 ft	250	None	No complications	Yes
9	49	M	32	78.41	APC2	Motor vehicle collision	300	I&D, repair scrotal laceration	No complications	Yes
10	91	M	25	51.27	APC2	Pedestrian struck by vehicle	100	None	No complications	Yes
11	54	M	28.4	55.61	APC2	Fall 15 ft	150	None	No complications	No
12	25	M	23.3	35.2	LC2	Fall 50 ft	300	None	No complications	No
13	35	M	28.3	51.3	VS	Motor vehicle collision	400	None	No complications	Yes
14	53	M	29.5	43.37	APC2	Motorcycle collision	200	None	No complications	No
15	73	M	31.6	82.98	LC3	Fall 15 ft	200	None	Mortality at 1 month	No
16	65	M	18.8	48.45	APC2	Equestrian	150	None	No complications	Yes
17	43	M	24.5	56.41	APC2	Fall 30 ft	200	None	No complications	Yes
18	64	M	31.4	60.54	APC2	Equestrian	200	None	No complications	No
19	64	M	30.5	54.54	APC3	Motorcycle collision	400	Extraperitoneal bladder repair	No complications	Yes
20	56	M	29.3	45.44	LC2	Motorcycle collision	150	None	No complications	Yes
21	38	M	23.9	47.36	APC2	Industrial accident	350	None	No complications	Yes
22	45	M	35.2	70.27	APC3	Jump/suicide attempt 30 ft	300	Extraperitoneal bladder repair	No complications	Yes
23	49	M	26.6	81.1	APC2	Motorcycle collision	250	None	No complications	Yes
24	51	M	33	54.31	APC2	Fall from mountain bike	200	None	No complications	Yes
25	61	M	32.7	61.24	APC2	Motorcycle collision	250	None	No complications	No

^a^Young and Burgess classification

*APC* anterior posterior compression, *LC* lateral compression, *VS* vertical shear, *BMI* body mass index

**Fig. 4:**
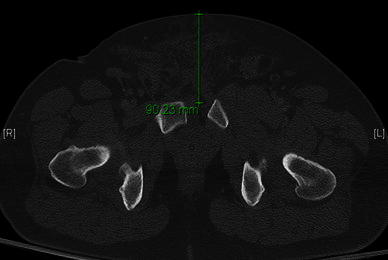
Axial CT image of an obese patient who had a complication following vertical midline skin incision. The distance between the pubic symphysis and skin was measured to be 9 cm

The average follow-up was 2.5 months (range 1–12 months). One patient died within a month of surgery as a result of their associated traumatic injuries. One patient developed a deep infection. This patient had a BMI of 34.3 and had a distance from skin to symphysis of 90.2 mm, which was the second largest BMI and greatest measured skin to symphysis depth in the series (Fig. 4). At the time of initial surgery, superficial skin blistering was noted to have occurred from the circumferential resuscitative sheet. A strong and foul odor was noted after the rectus abdominus fascia was split, although no bowel injury was ever identified. The urologic surgeons repaired his extra-peritoneal bladder rupture following pelvic ring fixation. The anterior pelvis was irrigated but fascial closure over the rectus was difficult due to the extent of the traumatic injury. On post-operative day 7, the midline pubic skin staples were prematurely removed. Skin healing was insufficient, resulting in wound dehiscence. The wound was subsequently debrided multiple times in the operating room and the bladder underwent repeat closure. A rectus abdominus rotational muscle flap was placed over the anterior bladder because of the deficient fascial layer and multiple debridements to isolate it from the symphyseal plate. Following a 6-week course of IV antibiotics and delayed skin grafting to the muscle flap, the patient recovered without further complication.

## Discussion

The pubic midline skin incision with surgical division of the rectus abdominus muscle fascia can be utilized as an effective alternative surgical approach to the pubic symphysis and anterior pelvic ring. Our results demonstrate that body habitus, severity of pelvic injury, and associated injuries do not preclude the use of this approach. Patient positioning, prep and drape as well as surgical suite setup remain the same when using the pubic midline or Pfannenstiel approach. Both exposures warrant complete perineal and pubic area skin preparation prior to surgery.

The pubic midline incision provides an essentially identical deep exposure to the anterior pelvic osteology as a transverse incision. The vertical skin incision does limit the extremes of peripheral exposure as the surgeon works laterally along the superior pubic ramus away from the midline. For surgeons familiar with the Pfannenstiel exposure, peripheral retraction of the rectus abdominus muscles and local pubic area soft tissues may seem more difficult via the pubic midline incision. In our series, the surgeons noted that while peripheral plate access was possible, the soft tissue tension via the pubic midline exposure affected the trajectory of peripheral plate screws, especially in the two patients with plates of ten holes. Otherwise, the pubic midline exposure did not change the surgical technique, clamp applications, reduction maneuvers, retractor placements, or implants used for symphyseal reduction and fixation. In 24 patients (96 %), the pubic midline wound healed without complication. The first patient in our series was the only one with a deep wound infection, and he had several related infection risks: (1) morbid obesity with a large abdominal pannus, (2) polytrauma with prolonged ICU stay, (3) pubic region skin blistering related to the circumferential pelvic sheeting during his resuscitation, (4) associated bladder injury and repair with failure and re-repair, (5) initial fascial repair failure, and (6) premature removal of skin staples. This infection rate is similar to that published on patients treated with the Pfannenstiel incision [2, 8, 9].

In addition to providing the orthopaedic surgeon with another option for surgical approach, the pubic midline incision and deep dissection are familiar to urological, vascular, gynecological, and general surgeons and provides excellent access to the bladder and bladder neck for repair of associated urologic injuries. This repair can be performed before or after fixation of the pubic symphysis and is dependent upon the location of the urologic injury and individual urologic surgeon preference, and the patient’s overall condition most importantly. When a general or trauma surgeon performs an exploratory laparotomy through an abdominal midline incision, the caudal extension of the exploratory laparotomy utilizes the same skin incision as the pubic midline. Therefore, the orthopaedic surgeon could use this single midline incision, rather than create a “T” incision at the low abdomen by joining the abdominal midline incision with a low transverse incision.

This study describes our experience with a pubic midline incision specifically for the surgical exposure, reduction and fixation of the pubic symphysis. The limitations of this study include its retrospective nature as well as the lack of comparison group. The decision by the treating surgeon to perform the low vertical midline incision was not based on set selection criteria, injury severity or body habitus. Our clinical follow-up was varied but continued through wound healing in all patients available for follow-up and averaged 2.5 months. Long-term follow-up was not included in the analysis, as the focus of this paper was on perioperative issues related to this technique. Therefore, long-term outcomes related to the patients’ injury and operation, such as deficits in musculoskeletal function as well as issues with the hardware, were not addressed in the review.

In conclusion, the low midline approach can be safely used as an alternative to the low transverse Pfannenstiel incision for the operative exposure, reduction and fixation of the anterior pelvic ring in most clinical scenarios. It provides another option for the orthopaedic surgeon and is quite familiar to certain other surgical sub-specialists and colleagues such as general, vascular, and urologic surgeons who may also be involved in the care of patients with pelvic ring injuries.

## References

[1] Matta JM, Saucedo T (1989). Internal fixation of pelvic ring fractures. Clin Orthop Relat Res.

[2] Papakostidis C, Kanakaris NK (2009). Pelvic ring disruptions: treatment modalities and analysis of outcomes. Int Orthop.

[3] Cole DJ, Bolhofner BR (1994). Acetabular fracture fixation via a modified Stoppa limited intrapelvic approach. Clin Orthop Relat Res.

[4] Ponsen K, Joosse P (2006). Internal fracture fixation using the Stoppa approach in pelvic ring and acetabular fractures: technical aspects and operative results. J Trauma.

[5] Letournel E (1993). The treatment of acetabular fractures through the ilioinguinal approach. Clin Orthop Relat Res.

[6] Burgess AR, Eastridge BJ, Young JW (1990). Pelvic ring disruptions: effective classification system and treatment protocols. J Trauma.

[7] Hirvensalo E, Lindahl J, Böstman O (1993). A new approach to the internal fixation of unstable pelvic fractures. Clin Orthop Relat Res.

[8] Cole JD, Blum DA, Ansel LJ (1996). Outcome after fixation of unstable posterior pelvic ring injuries. Clin Orthop Relat Res.

[9] Korovessis P, Baikousis A, Stamatakis M, Katonis P (2000). Medium- and long-term results of open reduction and internal fixation for unstable pelvic ring fractures. Orthopedics.

